# Anticancer and Anti-Inflammatory Properties of *Ganoderma lucidum* Extract Effects on Melanoma and Triple-Negative Breast Cancer Treatment

**DOI:** 10.3390/nu9030210

**Published:** 2017-02-28

**Authors:** Antonio Barbieri, Vincenzo Quagliariello, Vitale Del Vecchio, Michela Falco, Antonio Luciano, Nagoth Joseph Amruthraj, Guglielmo Nasti, Alessandro Ottaiano, Massimiliano Berretta, Rosario Vincenzo Iaffaioli, Claudio Arra

**Affiliations:** 1 Animal Facility Unit, Department of Research, National Cancer Institute “G. Pascale”, Via M. Semmola, 80131 Naples, Italy; vitale_84@hotmail.it (V.D.V.); michelafalco_89@libero.it (M.F.); a.luciano@istitutotumori.na.it (A.L.); c.arra@istitutotumori.na.it (C.A.); 2 Department of Abdominal Oncology, National Cancer Institute “G. Pascale”, Via M. Semmola, 80131 Naples, Italy; quagliariello.enzo@gmail.com (V.Q.); guglielmo.nasti@libero.it (G.N.); ale.otto@libero.it (A.O.); rv.iaffaioli@gmail.com (R.V.I.); 3 ASMO (Association for Multidisciplinary Studies in Oncology) and Mediterranean Diet, Piazza Nicola Amore 6, 80138 Naples, Italy; mberretta@cro.it; 4 Clinical, Experimental and Medical Sciences, Chair of Nephrology, Department of Cardio-Vascular Medicine, University of Study of Campania “Luigi Vanvitelli”, 81100 Caserta, Italy; amruthjon@gmail.com; 5 Department of Medical Oncology, National Cancer Institute, 33081 Aviano, Italy

**Keywords:** *Ganoderma lucidum*, melanoma, breast cancer, inflammation, cytokines, curcumin, cell viability

## Abstract

Among the most important traditional medicinal fungi, *Ganoderma lucidum* has been used as a therapeutic agent for the treatment of numerous diseases, including cancer, in Oriental countries. The aim of this study is to investigate the anti-inflammatory, anticancer and anti-metastatic activities of *Ganoderma lucidum* extracts in melanoma and triple-negative breast cancer cells. *Ganoderma lucidum* extracts were prepared by using common organic solvents; MDA-MB 231 and B16-F10 cell lines were adopted as cellular models for triple-negative breast cancer and melanoma and characterized for cell viability, wound-healing assay and measurement of cytokines secreted by cancer cells under pro-inflammatory conditions (incubation with lipopolysaccharide, LPS) and pretreatment with *Ganoderma lucidum* extract at different concentrations. Our study demonstrates, for the first time, how *Ganoderma lucidum* extracts can significantly inhibit the release of IL-8, IL-6, MMP-2 and MMP-9 in cancer cells under pro-inflammatory condition. Interestingly, *Ganoderma lucidum* extracts significantly also decrease the viability of both cancer cells in a time- and concentration-dependent manner, with abilities to reduce cell migration over time, which is correlated with a lower release of matrix metalloproteases. Taken together, these results indicate the possible use of *Ganoderma lucidum* extract for the therapeutic management of melanoma and human triple-negative breast cancer.

## 1. Introduction

*Ganoderma lucidum* is a bitter fungus with a glossy exterior and a woody texture [1]. It is commonly referred to as “lingzhi” in China, India and Japan, and it has been used in most Asian countries for the promotion of health and longevity for centuries [1,2]. Numerous pharmacological effects associated with lingzhi have been recorded, among which are immunomodulatory [3], anti-inflammatory [4], antiviral [5], antioxidative, antiaging and antitumor [6] properties. Several studies have shown that *Ganoderma lucidum* contains a wide range of bioactive compounds associated with the promotion of good health [1,2]. For all these reasons, *Ganoderma lucidum* is considered an interesting fungus, widely used in alternative medicine and proven to have numerous implications for use as a potential anticancer drug; however, further research needs to be done to quantify it for personalized medicine, especially in treating specific tumor diseases such as prostate adenocarcinoma (PCa) and other cancers [7]. The biological activities reported from preparations from *Ganoderma lucidum* are remarkable and are given the most emphasis herein as distinct from the structure/activity information. The metabolites consist of mainly polysaccharides and terpenoids, especially phenolic compounds Many of these biological compounds work against the major diseases of our time, and hence the present research is of great importance. The list of effects is huge, ranging from anti-cancer properties to relieving blockages of the bladder. However, the reports have not all been validated scientifically, and it is necessary to test every single pure component of the *Ganoderma lucidum* extracts to get convincing evidence about these features. It is a prime example of an ancient remedy being of great relevance to the modern era. We consider, as an assumption, that the therapeutic effects attributed to the fungus have been proven [1]. The next step is to produce some effective medicines, which may be hampered by problems of mass production. In recent years, our laboratories have been engaged in studying the effects of curcumin in several cancer cell lines [8,9,10,11]. Curcumin is a component of the Indian spice turmeric (*Curcuma longa*), known for its safety and low cost. Curcumin can selectively modulate multiple cell signaling pathways linked to inflammation and to survival, growth, invasion, angiogenesis, and metastasis of cancer cells. The aim of this study is to compare the effects of curcumin, considered as a positive control at a concentration of 10 µM, to treatment with *Ganoderma lucidum* extracts, testing different concentrations of these compounds and evaluating their effects on cell viability and pro-inflammatory cytokine secretion.

## 2. Materials and Methods

### 2.1. Cell Culture

Human mammary gland cancer cell line MDA-MB 231 and B16-F10 murine melanoma cell line, syngenic for C57Bl/6 mice, were obtained from the American Type Culture Collection (ATCC, Manassas, VA, USA). MDA-MB 231 cells were cultured in HIGH GLUCOSE DMEM (4.5 g/L Gibco) supplemented with 10% fetal bovine serum FBS (Gibco, Long Island, NY, USA) and 1% penicillin/streptomycin (P & S Sigma Aldrich, Milan, Italy). B16-F10 cells were morphologically authenticated and cultured in DMEM (Gibco), added with 10% FBS, 1% P & S and 1% l-Glutammine. All cell lines were maintained in humidified incubators at 37 °C under an atmosphere of 5% CO_2_.

### 2.2. Plant Materials

*Ganoderma lucidum* was provided by A. Arulappa Premkumar, Royappa nagar Tambaram, Chennai South India. The powder of dried *Ganoderma lucidum* was used for the preparation of mushroom extract. For the dimethyl sulfoxide (DMSO) extraction 3 gr of mushroom powder were mixed with 100 mL of DMSO and placed on a shaker for 24 h at room temperature. The solution was filtered with 3M™ 740 Cartridge and then placed on the rotary evaporator vacuum, for 15 min at 37 °C. Then the residue was dissolved in 100 mL of DMSO and stored at 4 °C for further analysis. The ethanolic extract was prepared using dried powder derived from fruiting body of *Ganoderma lucidum* (5.25 g), in ethanol 70% *v*/*v* (65 mL) for 10 h at room temperature, mixing with a blender. The suspension was filtered with Whatman^®^ #2 qualitative cellulose filter (Whatman, Maidstone, UK) and concentrated to 50 mL under vacuum; then the extract was stored at −20 °C for further analysis.

Curcumin powder, used for in vitro experiments, was obtained from Sigma Aldrich (Milan, Italy), it was dissolved in DMSO at room temperature and stored at −20 °C.

### 2.3. Proliferation Assay

The effect of *Ganoderma lucidum* on cell proliferation was determined by using TACS 3-(4,5-dimethylthiazol-2-yl)-2,5-diphenyltetrazolium bromide (MTT) cell proliferation assay (Trevigen, Githersburg). The cells, cultured in triplicate in a 96-well plate (2 × 104 per well), were incubated with DMSO or ethanolic *Ganoderma lucidum* extract, at different concentration. Then the cells were incubated for 24 h and 48 h at 37 °C under an atmosphere of 5% CO_2_. After this period the MTT solution was added to each well and incubated for 2 h at 37 °C. An extraction buffer (20% sodium dodecyl sulphate (SDS) and 50% dimethylformamide) was added, and the cells were incubated overnight at 37 °C. The absorbance of the cell suspension was measured at 570 nm using a microplate reader (DAS Technologies, Chantilly, VA, USA). This experiment was performed in triplicate, and the statistical analysis was performed to obtain the final values.

### 2.4. Wound-Healing Assay

MDA-MB 231 and B16-F10 cells were harvested from the exponential growth phase, washed twice by 1× Phosphate Buffered Saline (PBS) and resuspended in the respective medium, seeded at the density of 10^6^ cells per well into a six-multiwell plate and incubated at 37 °C in a humidified atmosphere of 5% CO_2_ for 24 h. Then, in each well, was made an horizontal slit with a white tip, in a central position; after a PBS 1× washing cells were incubated in the absence or presence of DMSO *Ganoderma lucidum* extract at different concentrations for 48 h. Cell migration on the slit of the confluent well was assessed at 0, 48 h, in each condition, by light microscopy reversed phase (Leica microsystems).

### 2.5. Cytokines Analysis

Cytokines production in cultural supernatant was determined by ELISA as recommended by the manufacturer (Sigma Aldrich, Milan, Italy). Briefly, MDA-MB 231 and B16-F10 cells (1.2 × 10^5^ cells/well) were seeded in 12-well plate in HIGH GLUCOSE DMEM (4.5 g/L Gibco) supplemented with 10% fetal bovine serum FBS (Gibco, Long Island, NY, USA) and 1% penicillin/streptomycin (P & S Sigma Aldrich) at 37 °C in a humidified 5% CO_2_ atmosphere. After pre-incubation for 24 h and starved in serum-free medium for 2.5 h, the cells were treated with or without 0.1 mL of a 2 and 5 mg/mL solution of DMSO *Ganoderma lucidum* extract for 30 min before exposure to LPS (50 ng/mL) for 12 h. The culture medium without any dilution was used to assay the cytokine production for four cytokines including IL-6, IL-8, MMP-2 and MMP-9 as recommended by the manufacturer. The sensitivity of this method was less than 10 pg/mL, and the assay can accurately detect cytokine since the range of 1–32,000 pg/mL.

### 2.6. Statistical Analysis

Results were taken from three independent experiments (*n* = 3) and data were expressed as means ± standard deviation (SD). Scatter diagram and histograms were drawn using Graph Pad Prim 5 (Graph Pad Software, La Jolla, CA, USA). Students’ *t*-test and one-way ANOVA followed by Bonferroni post hoc test were, respectively, applied to the comparison between two groups and multiple group comparison. A *p*-value < 0.05 was considered as statistically significant: * *p* < 0.05; ** *p* < 0.01; *** *p* < 0.001 versus respective control groups.

## 3. Results

### 3.1. Ganoderma lucidum Extracts Inhibit Cell Migration

**Figure 1:**
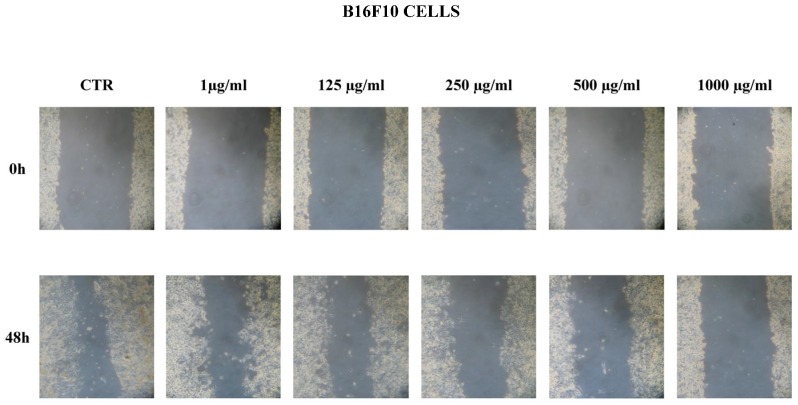
Wound-healing assay. B16F10 and MDA-MB231 cells were plated on a six-well plate. The cell layer was scratched and incubated with *Ganoderma lucidum* at different concentrations (1 µg/mL; 125 µg/mL; 250 µg/mL; 500 µg/mL; 1000 µg/mL) for 48 h. The images were captured by a Leica microsystem microscope with phase contrast. The experiment was repeated at least three times. CTR = control.

**Figure 2:**
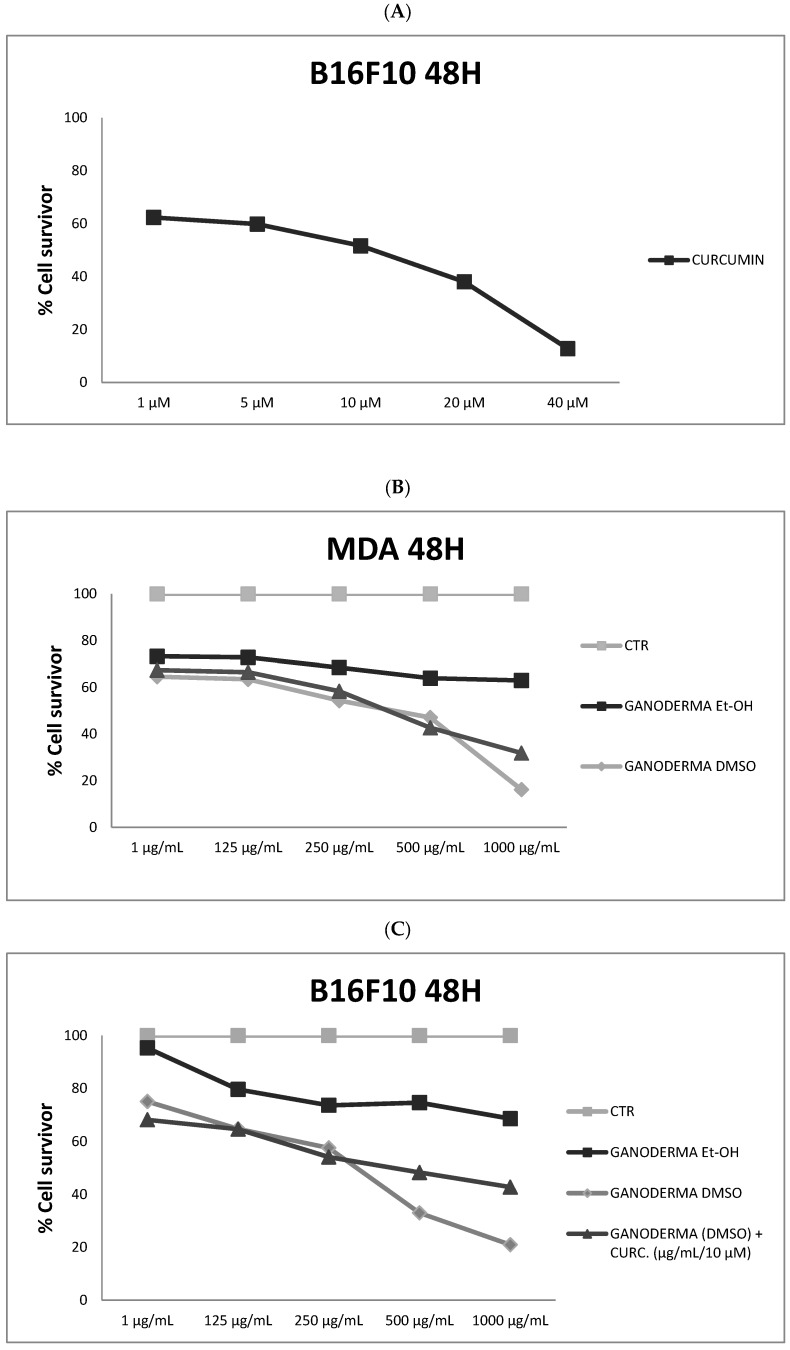
MTT assay. (**A**) Inhibitory effect of curcumin (IC_50_ = 10 µM); (**B**,**C**) MDA-MB231 and B16-F10 cells were plated on a 96-well plate and incubated with *Ganoderma lucidum* extracts at different concentrations (1 µg/mL; 125 µg/mL; 250 µg/mL; 500 µg/mL; 1000 µg/mL), and also combined with curcumin 10 µM for 48 h. The experiment was repeated at least three times. CTR = control; Et-OH = Ethanolic extract; DMSO = dimethyl sulfoxide extract; CURC = curcumin.

To assess the therapeutic properties of *Ganoderma lucidum*, we performed scratch and MTT assays on the murine melanoma cell line B16F10 and the triple-negative human breast cancer cell line MDA-MB231, respectively, because they are very aggressive with a fast duplication time (48 h). A qualitative scratch assay was performed at 48 h at different concentrations, respectively: 1, 125, 250, 500, 1000 µg/mL (Figure 1). Cell migration on the slit of the confluent wells, in both cell lines, was photographed at 0 and 48 h after, in each condition. As indicated in Figure 1, there was a remarkable dose-dependent inhibitory effect on cell migration that was already significant at the concentration of 250 µg/mL in both cell lines. All together, these data were confirmed by the semi-quantitative MTT assay. In our previous experience we found that the antitumoral activity of curcumin is significant at 10 µM (IC_50_); for this reason, we used this concentration for the following experiments in association with *Ganoderma lucidum* (Figure 2A). As showed in Figure 2B,C, ethanolic *Ganoderma lucidum* extract displayed a mild and not significant effect on the migration of both cell lines at 48 h. In the MDA-MB231 cell line DMSO, the *Ganoderma lucidum* extract showed an equivalent effect to *Ganoderma lucidum* plus curcumin treatment up to the concentration of 500 µg/mL (54% of cell viability); instead, at concentration of 1000 µg/mL, the effect of DMSO *Ganoderma lucidum* extract alone was stronger than the combined treatment of *Ganoderma lucidum* plus curcumin (Figure 2B). The B16F10 melanoma cell line showed a similar effect when treated with DMSO *Ganoderma lucidum* extract in combination with curcumin until the concentration of 250 µg/mL (57% of cell viability), while at the concentrations of 500 and 1000 µg/mL, the cells treated with only DMSO *Ganoderma lucidum* extract disclosed a lower viability than the combination (Figure 2C). 

### 3.2. Anti-Inflammatory Properties of Ganoderma lucidum Extracts

**Figure 3:**
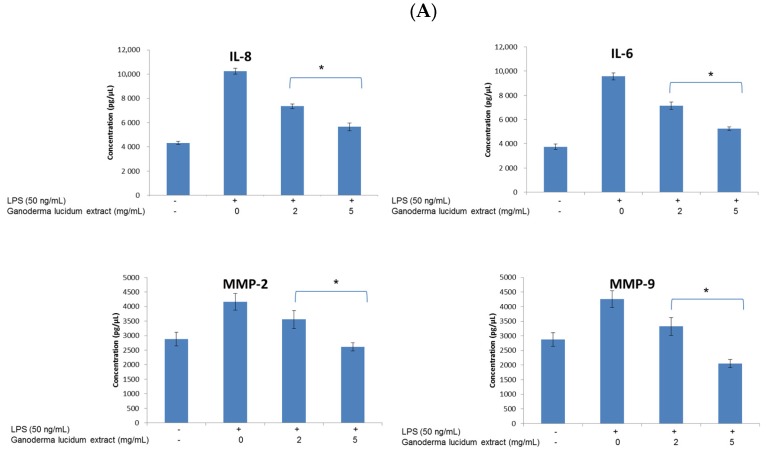

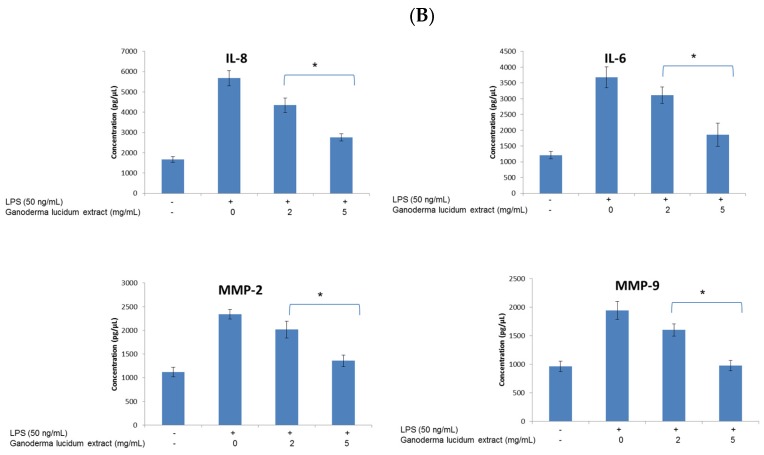
ELISA assay. The effect of DMSO *Ganoderma lucidum* extract on the concentration of IL-6, IL-8, MMP-2 and MMP-9. (**A**) Triple-negative breast cancer cells were treated with or without 2 and 5 mg/mL solution of DMSO *Ganoderma lucidum* extract for 30 min before exposure to lipopolysaccharide (50 ng/mL) for 12 h; (**B**) Melanoma cells were treated with or without 2 and 5 mg/mL solution of DMSO *Ganoderma lucidum* extract for 30 min before exposure to LPS (50 ng/mL) for 12 h. * (*p* ≤ 0.01).

To verify the biological activity of DMSO *Ganoderma lucidum* extract, we performed cytokine secretion studies. In particular, we evaluated the effects of treatment with *Ganoderma lucidum* on the release of IL-6, IL-8, MMP-2 and MMP-9 and other inflammatory cytokines promoting cancer. These experiments on melanoma and breast cancer cells were carried out under inflammatory conditions and they clearly highlighted the anti-inflammatory action of DMSO *Ganoderma lucidum* extract against LPS stimulation on cancer cells (Figure 3). More specifically, a pretreatment with LPS determined an increased secretion of measured cytokines and metalloproteases. This response may be due to the presence of the TLR4 receptor on the membrane of the melanoma and breast cancer cells, as was demonstrated [12,13]. Pretreatment with extract significantly decreased the levels of IL-8, IL-6, MMP-2 and MMP-9 in the breast cancer cells (Figure 3A). Moreover, *Ganoderma lucidum* extract at 2 mg/mL significantly reduced the magnitude of the cellular levels of IL-8, IL-6, MMP-2 and MMP-9 by approximately 28%, 25%, 15% and 22%, respectively, compared to untreated cells (*p* ≤ 0.01). Interestingly, *Ganoderma lucidum* extract at 5 mg/mL reduced the magnitude of the cellular levels of IL-8, IL-6, MMP-2 and MMP-9 by approximately 45%, 44%, 38% and 52%, respectively, compared to untreated cells (*p* ≤ 0.01). Relative to the melanoma cells, in this case pretreatment with extract also significantly decreased the levels of IL-8, IL-6, MMP-2 and MMP-9 (Figure 3B). Specifically, *Ganoderma lucidum* extract at 2 mg/mL always reduced the magnitude of the cellular levels of IL-8, IL-6, MMP-2 and MMP-9 in a significant manner, by approximately 23%, 16%, 14% and 18%, respectively, compared to untreated cells (*p* ≤ 0.01). Interestingly, *Ganoderma lucidum* extract at 5 mg/mL reduced the magnitude of the cellular levels of IL-8, IL-6, MMP-2 and MMP-9 by approximately 52%, 50%, 42% and 50%, respectively, compared to untreated cells (*p* ≤ 0.01).

## 4. Discussion

*Ganoderma lucidum* is a mushroom rich with bioactive compounds, among which are fatty acids, nucleosides, amino acids, proteins, peptides, alkaloids, steroids, enzymes, triterpenoids and polysaccharides. Among all of these, triterpenoids and polysaccharides represent the main constituents contributing to its anticancer functions [14,15]. Many *Ganoderma lucidum* polysaccharides (GLPS) exert immune-modulating functions through activating the expression of cytokines associated with inflammatory response (such as interleukin-1, interleukin-6, and tumor necrosis factor-α) or anti-tumor activity (such as interferon-γ and tumor necrosis factor-α) [16]. Even if some authors indicate a direct cytotoxicity of GLPS on cancer cells [17], the anticancer functions of GLPS are, however, still generally believed to be closely associated with their immune-stimulating effects [1]. Growing clinical evidence suggests the important role of pro-inflammatory cytokines during tumor development. Specifically, many cytokines were reported to act as pro-inflammatory factors both in melanoma and triple-negative breast cancer: as an example, recent studies have shown that several cytokines are produced by cancer cells, or by the tumor microenvironment, in order to increase their progression and survival. Specifically, the association between interleukin-6 (IL-6) and the pathogenesis of melanoma and breast cancer was studied [18,19]. Increased IL-6 expression has been related to an advanced disease stage and decreased survival in breast and melanoma cancer patients [20,21]. The release of IL-6 is biologically related to an induction of tumor cell proliferation and inhibition of cellular apoptosis through the involvement of Janus kinases (JAKs) and signal transducer and activator of transcription 3 (STAT3). Similarly, interleukin-8 (IL-8), a chemokine with a defining CXC amino acid motif, is characterized by important tumorigenic and pro-angiogenic properties in both cancers and it is associated with poor prognosis [22,23]. Moreover, IL-8 over-expression is involved in the proliferation, metastasis, angiogenesis and sensitivity to chemotherapeutics in breast cancer, primarily through the activation of AKT and MAPK signaling which results in the increased activation of NF-κB [12,13]. Melanoma and breast cancer cells are able to release several interleukins such as IL-8 and IL-6 under pro-inflammatory conditions [24,25]. Our data demonstrate that DMSO *Ganoderma lucidum* extract exerts anticancer effects on cell migration and anti-inflammatory effects after stimulation with LPS. All together, our data indicate that the *Ganoderma lucidum* extract could represent a new strategy for anticancer treatment and anti-inflammatory properties due to the presence of a wide range of compounds which exert beneficial effects on health and potentiate the immune response. 

## 5. Conclusions

These results suggest that the complex of bioactive compounds derived from *Ganoderma lucidum* extract, presumably a mix of polyphenols and carbohydrate-flavonoid complexes, can be beneficially exploited in anticancer and anti-inflammatory therapies against melanoma and breast cancer. However, further biological studies are required and are currently being carried out in order to characterize the molecular mechanisms of action of the extracts and to elucidate the cellular pathways involved. More specifically, molecular biology studies are currently ongoing in our research group in order to identify the panel of genes influenced by the incubation of cancer cells with the extracts. Therefore, the use of *Ganoderma lucidum* extracts may open a new therapeutic opportunity in the treatment and management, during chemotherapy, of melanoma and breast cancer, especially considering its strong anti-inflammatory and anti-metastatic actions which can lead to it being used as a new therapeutic tool in cancer management.
